# KIF11 UFMylation Maintains Photoreceptor Cilium Integrity and Retinal Homeostasis

**DOI:** 10.1002/advs.202400569

**Published:** 2024-04-26

**Authors:** Jie Ran, Guizhi Guo, Sai Zhang, Yufei Zhang, Liang Zhang, Dengwen Li, Shian Wu, Yusheng Cong, Xiaohong Wang, Songbo Xie, Huijie Zhao, Hongbin Liu, Guangshuo Ou, Xueliang Zhu, Jun Zhou, Min Liu

**Affiliations:** ^1^ Center for Cell Structure and Function Shandong Provincial Key Laboratory of Animal Resistance Biology Haihe Laboratory of Cell Ecosystem College of Life Sciences Shandong Normal University Jinan 250014 China; ^2^ Department of Genetics and Cell Biology State Key Laboratory of Medicinal Chemical Biology College of Life Sciences Nankai University Tianjin 300071 China; ^3^ Key Laboratory of Aging and Cancer Biology of Zhejiang Province Institute of Aging Research School of Medicine Hangzhou Normal University Hangzhou 310036 China; ^4^ Department of Pharmacology Tianjin Key Laboratory of Inflammation Biology School of Basic Medical Sciences Tianjin Medical University Tianjin 300070 China; ^5^ Center for Reproductive Medicine Cheeloo College of Medicine Key Laboratory of Reproductive Endocrinology of Ministry of Education Shandong University Jinan 250014 China; ^6^ Tsinghua‐Peking Center for Life Sciences Ministry of Education Key Laboratory for Protein Science School of Life Sciences Tsinghua University Beijing 100084 China; ^7^ State Key Laboratory of Cell Biology CAS Center for Excellence in Molecular Cell Science Shanghai Institute of Biochemistry and Cell Biology Chinese Academy of Sciences Shanghai 200031 China; ^8^ Laboratory of Tissue Homeostasis Haihe Laboratory of Cell Ecosystem Tianjin 300462 China

**Keywords:** cilium, KIF11, photoreceptor, ubiquitination, UFMylation

## Abstract

The photoreceptor cilium is vital for maintaining the structure and function of the retina. However, the molecular mechanisms underlying the photoreceptor cilium integrity and retinal homeostasis are largely unknown. Herein, it is shown that kinesin family member 11 (KIF11) localizes at the transition zone (connecting cilium) of the photoreceptor and plays a crucial role in orchestrating the cilium integrity. KIF11 depletion causes malformations of both the photoreceptor ciliary axoneme and membranous discs, resulting in photoreceptor degeneration and the accumulation of drusen‐like deposits throughout the retina. Mechanistic studies show that the stability of KIF11 is regulated by an interplay between its UFMylation and ubiquitination; UFMylation of KIF11 at lysine 953 inhibits its ubiquitination by synoviolin 1 and thereby prevents its proteasomal degradation. The lysine 953‐to‐arginine mutant of KIF11 is more stable than wild‐type KIF11 and also more effective in reversing the ciliary and retinal defects induced by KIF11 depletion. These findings identify a critical role for KIF11 UFMylation in the maintenance of photoreceptor cilium integrity and retinal homeostasis.

## Graphical abstract

The photoreceptor cilium integrity is vital for maintaining retinal homeostasis. This study shows that the stability of kinesin family member 11 (KIF11) is modulated by an interplay between its UFMylation and ubiquitination, which plays a crucial role in orchestrating the cilium integrity. Their findings uncover an important role for KIF11 UFMylation in the maintenance of photoreceptor cilium integrity and retinal homeostasis.

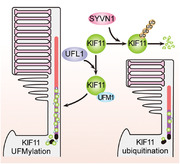

## 1 Introduction

The vertebrate retina is a layered structure with a wide variety of cells that form distinct circuits working parallel to produce a complex visual output.^[^ ^1^ ^]^ The structure of the retina is conserved across vertebrates and includes three major layers: the outmost retinal pigment epithelial (RPE) layer, the photoreceptor‐containing outer nuclear layer (ONL), and the inner nuclear layer (INL) which contains Müller cells and bipolar cells. This intricate arrangement is highly prone to damage, leading to disruptions in retinal homeostasis and contributing to an array of retinal diseases, such as diabetic retinopathy, retinitis pigmentosa, and age‐related macular degeneration.^[^ ^2^, ^3^, ^4^ ^]^ Among these retinal diseases, photoreceptor degeneration is the leading cause of blindness.^[^ ^5^ ^]^

Photoreceptors are polarized neurons with very specific subcellular compartmentalization mediating photosensation.^[^ ^6^ ^]^ There are two major groups of photoreceptors, cones and rods. Cones respond to bright light, mediate color vision, and permit high resolution of visual images, while rods function only under low‐light conditions and can respond to single light quanta, being more sensitive than cones.^[^ ^7^, ^8^ ^]^ Both cones and rods contain an outer segment, the site for photon capture that initiates vision; an inner segment, which houses the biosynthetic machinery; and a synaptic terminal for signal transmission to downstream neurons.^[^ ^9^ ^]^ The elaborate architecture of the outer segment comprises hundreds of stacked membranous discs and a microtubule‐based ciliary axoneme that extends from the basal body of the inner segment.^[^ ^6^ ^]^ This specialized primary cilium is finely adapted to transduce signals and transport cargoes to the outer segment, but its organization also makes it vulnerable to dysfunction.

Photoreceptor cilium dysfunction has been implicated in various eye diseases, as gene mutations that affect ciliary structure or trafficking have been shown to cause photoreceptor degeneration and visual loss.^[^ ^10^ ^]^ There is accumulating evidence that targeting photoreceptor cilia or ciliary proteins may represent a promising treatment for retinal degeneration.^[^ ^11^, ^12^ ^]^ Kinesin family member 11 (KIF11), which is also known as Eg5, kinesin‐5, or kinesin spindle protein,^[^ ^13^ ^]^ is a crucial ciliary regulator localized at the basal body of the primary cilium.^[^ ^14^, ^15^ ^]^ Additionally, a number of mutations in the *KIF11* gene have been identified in patients with retinal diseases.^[^ ^16^, ^17^ ^]^ In this study, we uncover an important role for KIF11 UFMylation in the maintenance of photoreceptor cilium integrity and retinal homeostasis, suggesting a novel avenue for the treatment of retinal diseases.

## 2 Results

### 2.1 KIF11 Is Required for the Maintenance of Photoreceptor Cilium Integrity and Retinal Homeostasis

**Figure 1:**
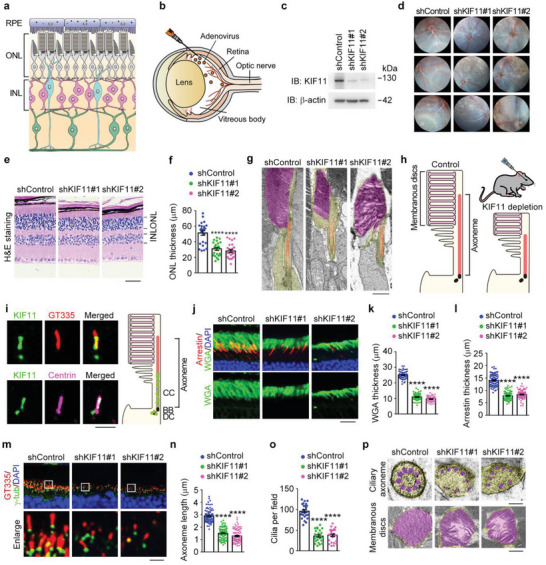
Impairment of photoreceptor ciliary structure and retinal homeostasis in mice intravitreally injected with KIF11‐shRNA adenoviruses. a) A schematic illustration of the retina, primarily composed of the RPE (retinal pigment epithelium), ONL (outer nuclear layer), and INL (inner nuclear layer). b) A schematic illustration of intravitreal injection of control or KIF11‐shRNA adenoviruses. c) Immunoblot analysis of KIF11 and β‐actin in retinas of control or KIF11‐shRNA adenovirus‐injected mice. d) Images of fundus photography in control or KIF11‐shRNA adenovirus‐injected mice. e,f) Photomicrographs (e) and quantification (f) of the retinal histology assessed by H&E staining in control or KIF11‐shRNA adenovirus‐injected mice (*n* = 24 eyes from 12 mice of three independent experiments). Scale bar, 30 µm. g,h) Transmission electron microscopy images of the longitudinal sections (g) and schematic illustration (h) of photoreceptors in control or KIF11‐shRNA adenovirus‐injected mice. Scale bar, 1 µm. i) Immunofluorescence staining of mouse retinas stained with antibodies against KIF11, polyglutamylated tubulin (GT335), and centrin. CC, connecting cilium; BB, basal body; DC, daughter centriole. Scale bars, 2 µm. j–l) Immunofluorescence images (j) and quantifications of membranous disc thickness in rods (k) and cones (l) from control or KIF11‐shRNA adenovirus‐injected mice (*n* = 60 fields from 12 mice of three independent experiments). Scale bar, 20 µm. m–o) Immunofluorescence images (m) and quantification of ciliary axoneme length (n, *n* = 80 fields from 12 mice of three independent experiments) and ciliary density (o, *n* = 24 eyes from 12 mice of three independent experiments) in retinas from control or KIF11‐shRNA adenovirus‐injected mice. Scale bar, 3 µm. (p) Transmission electron microscopy images of the cross sections of ciliary axonemes and membranous discs in control or KIF11‐shRNA adenovirus‐injected mice. Scale bars, 0.1 µm (upper) and 0.5 µm (bottom). Data are presented as mean ± SEM. ****p < 0.0001.

The retina comprises three distinct cell body layers, separated by two synaptic or plexiform layers (**Figure** **1**a). To investigate the potential involvement of KIF11 in retinal homeostasis, mice were intravitreally injected with adenoviruses encoding small hairpin RNAs (shRNAs) targeting this protein (Figure 1b). The level of retinal KIF11 was dramatically decreased upon injection with KIF11‐shRNA adenoviruses (Figure 1c), as intravitreal injection has been demonstrated to efficiently deliver adenovirus particles to photoreceptors (Figure S1a, Supporting Information).^[^ ^12^, ^18^ ^]^ Fundus photography, a clinical tool frequently used for the evaluation of retinopathies,^[^ ^19^ ^]^ was performed to analyze retinal structures in these mice. We observed drusen‐like deposits (DLDs) throughout the retina in KIF11‐depleted retinas (Figure 1d; Figure S1b, Supporting Information).

To determine whether KIF11 plays a role in regulating retinal structure, we assessed retinal morphology by performing histopathological analysis of hematoxylin and eosin (H&E)‐stained sections. We found that the thickness of retinal ONL was significantly reduced upon KIF11 depletion (Figure 1e,f). However, no discernible changes were detected in the RPE or INL layers in retinas injected with KIF11‐shRNA adenoviruses, indicating the specific disruption of the photoreceptor. To elucidate the exact role of KIF11 in the photoreceptor, we examined the longitudinal sections of photoreceptors with transmission electron microscopy. We found that the membranous discs, where the visual processes are initiated, were destroyed upon KIF11 depletion (Figure 1g). Additionally, the ciliary axoneme, which is responsible for the transport of membranous disc proteins, including the opsin family of proteins, was significantly shortened (Figure 1g,h). Our previous results and those of others demonstrated the localization of KIF11 at the basal body of primary cilia.^[^ ^14^, ^15^ ^]^ However, in the retinal photoreceptors, KIF11 was found to localize at the transition zone (connecting cilium) and daughter centriole, as revealed by immunostaining of KIF11 together with polyglutamylated tubulin, a ciliary axoneme marker,^[^ ^20^ ^]^ or centrin, a marker for the connecting cilium and basal body (originating from the mother centriole) in the photoreceptor^[^ ^21^ ^]^ (Figure 1i). These results suggest that KIF11 regulates ciliary axoneme assembly, which may in turn affect the integrity of photoreceptor cilia and the overall homeostasis of the retina.

To test this possibility, the membranous discs were stained with wheat germ agglutinin (WGA) and the anti‐arrestin antibody to visualize changes in rod and cone photoreceptors,^[^ ^22^, ^23^ ^]^ respectively. Immunofluorescence microscopy revealed that the membranous discs of both rods and cones were severely disrupted in KIF11‐depleted mouse retinas (Figure 1j–l). We next examined the ciliary axonemes of photoreceptors. Immunostaining of polyglutamylated tubulin and γ‐tubulin, a ciliary basal body marker, showed that the length and density of photoreceptor ciliary axonemes were remarkably reduced upon KIF11 depletion (Figure 1m–o). Similar results were achieved by immunostaining of ADP ribosylation factor like GTPase 13B (Arl13b),^[^ ^24^ ^]^ another well‐known ciliary marker (Figure S1c–e, Supporting Information). To confirm these observations, we examined the cross sections of photoreceptors by transmission electron microscopy. Both membranous discs and ciliary axonemes in KIF11‐depleted mice exhibited varying degrees of abnormality (Figure 1p; Figure S1f, Supporting Information). Collectively, these results suggest that KIF11 is required for the maintenance of photoreceptor cilium integrity and retinal homeostasis.

### 2.2 UFL1 Interacts with and UFMylates KIF11

**Figure 2:**
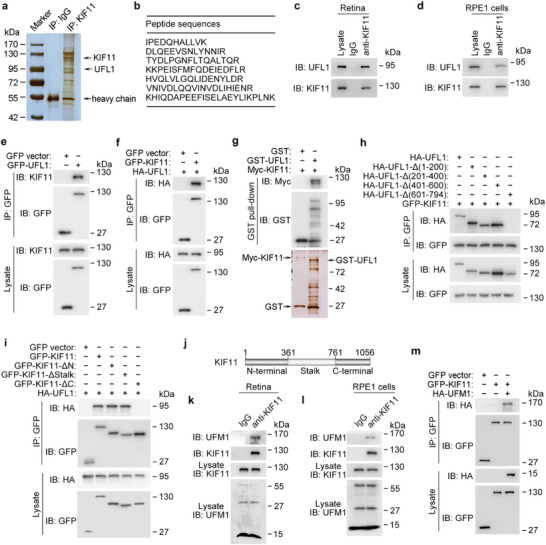
UFL1 interacts with KIF11 both in vivo and in vitro. a,b) Silver staining of the proteins immunoprecipitated from mouse retinal lysates with the anti‐KIF11 antibody (a), and sequences of the UFL1 peptides identified by mass spectrometry (b). c,d) Immunoprecipitation and immunoblotting showing the interaction between endogenous UFL1 and KIF11 in the retina and RPE1 cells. e,f) Immunoprecipitation and immunoblotting showing the interaction of GFP‐UFL1 with endogenous KIF11 (e) and GFP‐KIF11 with HA‐UFL1 (f) in HEK293T cells. g) GST pull‐down showing the interaction of purified GST‐UFL1 with purified Myc‐KIF11 protein. h,i) Identification of the domains mediating the interaction between UFL1 and KIF11 using various deletion constructs of HA‐UFL1 (h) or GFP‐KIF11 (i). j) Schematic diagram of KIF11. k,l) UFMylation of endogenous KIF11 was analyzed by immunoprecipitation with the anti‐KIF11 antibody followed by immunoblotting with the anti‐UFM1 antibody in the mouse retina (k) and RPE1 cells (l). m) UFMylation of KIF11 was analyzed by immunoprecipitation with the anti‐GFP antibody followed by immunoblotting in cells transfected with HA‐UFM1 and GFP‐KIF11 or GFP vector.

To explore the molecular mechanisms regulating the retinal function of KIF11, we immunoprecipitated this protein from mouse retinal lysates. Mass spectrometric analysis of proteins present in the KIF11 immunoprecipitate revealed that ubiquitin‐fold modifier 1 (UFM1)‐specific ligase 1 (UFL1), the sole ligase mediating UFMylation,^[^ ^25^ ^]^ was a KIF11‐interacting protein (**Figure** **2**a,b; and Table S1, Supporting Information). Additional immunoprecipitation experiments confirmed the interaction of UFL1 with KIF11 in retinal lysates and human retinal pigment epithelial 1 (RPE1) cells (Figure 2c,d; Figure S2a, b, Supporting Information). In addition, immunoprecipitation revealed that GFP‐UFL1 interacted with the endogenous KIF11, and GFP‐KIF11 interacted with HA‐UFL1 in human embryonic kidney 293T (HEK293T) cells (Figure 2e,f). Meanwhile, GST pull‐down assays with recombinant GST‐UFL1 and Myc‐KIF11 confirmed the interaction of UFL1 with KIF11 in vitro (Figure 2g). Using a series of truncated UFL1 and KIF11 constructs, we found that the 601–794 aa region of UFL1 was important for its interaction with KIF11 and that the carboxy‐terminal region of KIF11 was required for its interaction with UFL1 (Figure 2h–j). Strikingly, we also observed the localization of both KIF11 and UFL1 at the basal body of the primary cilium (Figure S2c, Supporting Information).

**Figure 3:**
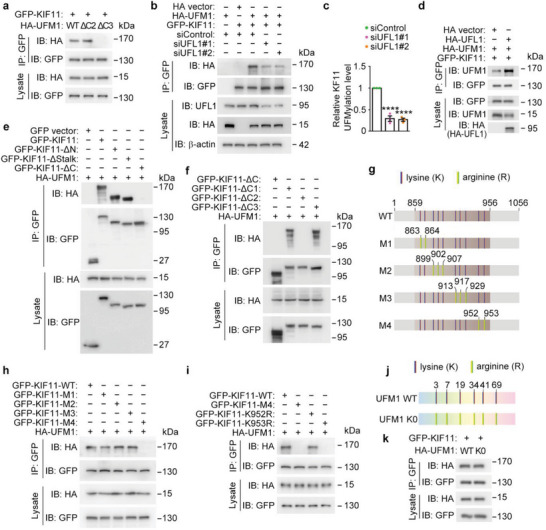
UFL1 mediates KIF11 UFMylation. a) KIF11 UFMylation was analyzed by immunoprecipitation with the anti‐GFP antibody followed by immunoblotting with the anti‐HA antibody in HEK293T cells transfected with GFP‐KIF11 and HA‐UFM1 or its mutants lacking two or three amino acids at the carboxy‐terminus (ΔC2 or ΔC3). b–d) Analysis of KIF11 UFMylation in cells transfected with GFP‐KIF11 with or without UFL1 siRNAs (b, c) and in cells transfected with GFP‐KIF11 together with HA vector or HA‐UFL1 (d). Relative KIF11 UFMylation level were determined by densitometry (c, *n* = 3 independent experiments). e) Analysis of the UFMylation of full‐length KIF11 and its deletion mutants in cells transfected with HA‐UFM1 together with GFP vector or GFP‐KIF11 or its deletion mutants. f) Analysis of the UFMylation of deletion mutants of the KIF11 carboxy‐terminal region. g–i) Identification of the KIF11 UFMylation site by immunoprecipitation with the anti‐GFP antibody followed by immunoblotting with the anti‐HA antibody in cells transfected with HA‐UFM1 and KIF11 lysine to arginine mutants. j,k) Analysis of KIF11 UFMylation by immunoprecipitation with the anti‐GFP antibody followed by immunoblotting with the anti‐HA antibody in HEK293T cells transfected with GFP‐KIF11 and HA‐UFM1 wild‐type or its lysine‐less (K0) mutant. Data are presented as mean ± SEM. ****p < 0.0001.

To determine whether KIF11 is a substrate of UFL1‐mediated UFMylation, endogenous UFM1 or KIF11 was immunoprecipitated from the mouse retina and RPE1 cells. Indeed, we found that endogenous KIF11 underwent remarkable UFMylation (Figure 2k,l; Figure S2d,e, Supporting Information). In addition, we coexpressed GFP‐KIF11 and HA‐UFM1 or UFM1 mutants in HEK293T cells. Immunoprecipitation experiments showed that wild‐type UFM1 and an active form of UFM1 with an exposed carboxy‐terminal glycine 83 residue (ΔC2), but not an inactive form of UFM1 lacking the carboxy‐terminal glycine 83 (ΔC3),^[^ ^26^ ^]^ could conjugate with KIF11 (Figures 2m and **3**a). Consistently, knockdown of UFL1 with two different siRNAs efficiently decreased the UFMylation of KIF11 (Figure 3b,c). Conversely, overexpression of HA‐UFL1 significantly increased the level of KIF11 UFMylation (Figure 3d). Taken together, these results indicate that UFL1 promotes the UFMylation of KIF11.

### 2.3 KIF11 UFMylation at K953 Enhances Its Stability

To identify the UFMylation site in KIF11, we coexpressed HA‐UFM1 and GFP‐KIF11 or its deletion mutants. Immunoprecipitation experiments demonstrated that the carboxy‐terminal region of KIF11 was required for its UFMylation (Figure 3e). To map the essential residue(s) for KIF11 UFMylation, we generated three deletion mutants for the carboxy‐terminal region of KIF11. Deletion of the C2 region (860‐957 aa), but not the C1 region (762‐859 aa) or C3 region (958‐1056 aa), of KIF11 significantly reduced its UFMylation (Figure 3f), indicating potential UFMylation site(s) in the C2 region of KIF11. As there are 10 lysine residues in the C2 region of KIF11, we generated lysine‐to‐arginine (KR) mutations in various combinations (Figure 3g). Of these, only the K952/953R mutations (lysines 952 and 953 replaced by arginines) restrained the UFMylation of KIF11 (Figure 3h), indicating the occurrence of KIF11 UFMylation at these two lysine residues. Analysis of individual KR mutants further revealed that the K953R mutation, but not the K952R mutation, completely abolished KIF11 UFMylation (Figure 3i). In addition, we found that the UFMylation of KIF11 by wild‐type UFM1 was similar to that by the lysine‐less (K0) mutant of UFM1 (Figure 3j,k), which can only produce mono‐UFM1 modification.^[^ ^27^ ^]^ Based on these results, we conclude that KIF11 undergoes mono‐UFMylation at the K953 residue.

**Figure 4:**
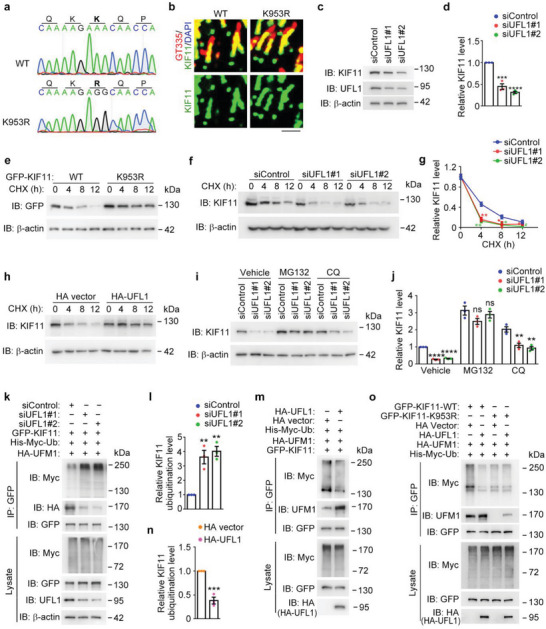
KIF11 UFMylation prevents its ubiquitination and proteasomal degradation. a) Sanger sequencing verification of the mutation in the *Kif11* gene locus. b) Immunofluorescence staining of KIF11 and GT335 in retinas from wild‐type or K953R knock‐in mice. Scale bar, 2 µm. c,d) Immunoblot analysis of KIF11, UFL1, and β‐actin in cells transfected with control or UFL1 siRNAs. Relative KIF11 levels were determined by densitometry (*n* = 3 independent experiments). e) The protein stability of wild‐type or K953R mutant KIF11 was examined by immunoblotting in cells transfected with GFP‐KIF11 wild‐type or the K953R mutant and treated with 20 µg mL^−1^ cycloheximide (CHX) for the indicated time. f–h) KIF11 protein stability was examined by immunoblotting in cells transfected with control or UFL1 siRNAs (f, g) or transfected with HA vector or HA‐UFL1 plasmids (h), and then treated with 20 µg mL^−1^ cycloheximide for the indicated time. Relative KIF11 levels were determined by densitometry (*n* = 3 independent experiments). i,j) Immunoblot analysis of KIF11 and β‐actin in cells transfected with control or UFL1 siRNAs and treated with MG132 (5 mm), chloroquine (CQ, 10 mm), or vehicle (DMSO) for 12 h. Relative KIF11 ubiquitination levels were determined by densitometry (*n* = 3 independent experiments). k–n) Ubiquitination and UFMylation of KIF11 were analyzed by immunoprecipitation with the anti‐GFP antibody followed by immunoblotting with anti‐Myc and anti‐HA or anti‐UFM1 antibodies in cells transfected with HA‐UFM1, His‐Myc‐ubiquitin, or GFP‐KIF11 together with control or UFL1 siRNAs (k) or with HA vector or HA‐UFL1 plasmids (m), and the relative KIF11 ubiquitination level were determined by densitometry (l and n, *n* = 3 independent experiments). o) Analysis of the UFMylation of KIF11 wild‐type or K953R mutant by immunoprecipitation with the anti‐GFP antibody followed by immunoblotting with anti‐Myc and anti‐UFM1 antibodies in cells transfected with HA‐UFM1, His‐Myc‐ubiquitin, GFP‐KIF11, or GFP‐KIF11‐K953R together with HA vector or HA‐UFL1. Data are presented as mean ± SEM. *p < 0.5, **p < 0.01, ***p < 0.001, ****p < 0.0001; ns or #, not significant.

To investigate whether UFL1‐mediated KIF11 UFMylation impacts its localization or protein level, we generated knock‐in mice harboring the K953R mutation by introducing the corresponding nucleotide mutations (c.A2858/2859G) into the mouse *Kif11* gene locus using the CRISPR/Cas9 technology. Sanger sequencing was performed to confirm the introduction of the mutations (**Figure** **4**a). Immunofluorescence microscopy revealed that the KIF11 K953R mutant still localized to the connecting cilium and the daughter centriole of photoreceptors, indicating that UFMylation did not affect the localization of KIF11 within photoreceptors (Figure 4b; Figure S3a,b, Supporting Information). Additionally, we observed that the photoreceptor ciliary length and intensity of K953R mice did not exhibit obvious changes, compared to wild‐type mice (Figure 4b; Figure S3c,d, Supporting Information). Subsequently, we examined the effect of UFL1 siRNAs on KIF11 protein and mRNA levels. We found that knockdown of UFL1 significantly decreased the KIF11 protein level but not its mRNA level (Figure 4c,d; Figure S3e, Supporting Information). These results prompted us to assess whether KIF11 UFMylation affects its stability. Thus, we treated cells with the protein synthesis inhibitor cycloheximide and subsequently examined the half‐life of KIF11. We found that the K953R mutant had a much longer half‐life than wild‐type KIF11 (Figure 4e; Figure S3f, Supporting Information). Furthermore, the half‐life of KIF11 was much shorter in UFL1‐depleted cells and longer in cells overexpressing UFL1, compared to cells transfected with empty vector or the control siRNA (Figure 4f–h; Figure S3g, Supporting Information). These results suggest that KIF11 UFMylation at K953 enhances its stability.

### 2.4 KIF11 UFMylation Blocks Its Degradation by the Ubiquitin‐Proteasome Pathway

We next studied the mechanism by which UFL1‐mediated UFMylation of KIF11 increases its stability. We treated cells with the proteasome inhibitor MG132 or the autophagy inhibitor chloroquine (CQ). We found that UFL1 depletion‐induced KIF11 degradation was significantly prevented by MG132 and only slightly prevented by CQ (Figure 4i,j), suggesting that UFMylation may inhibit KIF11 degradation through the ubiquitin‐proteasome pathway. Indeed, the ubiquitination of KIF11 was dramatically increased upon UFL1 knockdown (Figure 4k,l). Conversely, increase of KIF11 UFMylation by overexpressing UFL1 could reduce the ubiquitination of KIF11 (Figure 4m,n), indicating that KIF11 UFMylation at K953 may antagonize its ubiquitination. To test this possibility, we examined the ubiquitination of the K953R mutant. We found that the ubiquitination of this mutant was obviously decreased compared with that of wild‐type KIF11 and that this decreased ubiquitination was not affected by overexpression of UFL1 (Figure 4o). These results suggest that KIF11 UFMylation at K953 increases its stability by blocking its degradation by the ubiquitin‐proteasome pathway.

**Figure 5:**
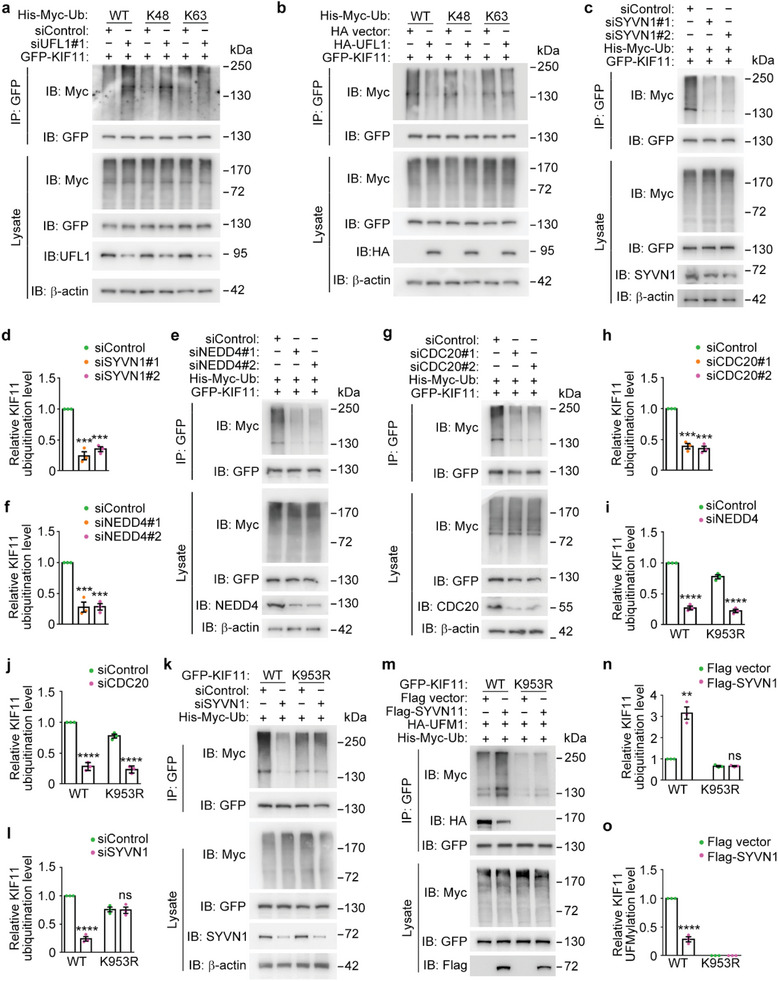
KIF11 UFMylation at K953 inhibits its ubiquitination by SYVN1. a) Analysis of KIF11 ubiqutination by immunoprecipitation with the anti‐GFP antibody followed by immunoblotting with the anti‐Myc antibody in cells transfected with GFP‐KIF11 and His‐Myc‐ubiquitin wild type or different mutants, together with control or UFL1 siRNAs. b) Analysis of KIF11 ubiqutination by immunoprecipitation with the anti‐GFP antibody followed by immunoblotting with the anti‐Myc antibody in cells transfected with GFP‐KIF11 and His‐Myc‐ubiquitin wild type or different mutants, together with HA vector or HA‐UFL1. c,d) Analysis of KIF11 ubiquitination by immunoprecipitation with the anti‐GFP antibody followed by immunoblotting with the anti‐Myc antibody in cells transfected with GFP‐KIF11 and His‐Myc‐ubiquitin together with control or SYVN1 siRNAs (c). Relative KIF11 ubiqutination levels were determined by densitometry (d, *n* = 3 independent experiments). e–h) Analysis of KIF11 ubiquitination by immunoprecipitation with the anti‐GFP antibody followed by immunoblotting with the anti‐Myc antibody in cells transfected with GFP‐KIF11 and His‐Myc‐ubiquitin together with control, NEDD4 siRNAs (e), or CDC20 siRNAs (g). Relative KIF11 ubiquitination levels were determined by densitometry (f and h, *n* = 3 independent experiments). i,j) Quantification of the relative KIF11 ubiquitination in HEK293T cells transfected with GFP‐KIF11 and His‐Myc‐ubiquitin, together with control, NEDD4 (i), or CDC20 (j) siRNAs (*n* = 3 independent experiments). k–n) Analysis of the ubiquitination of KIF11 wild‐type or K953R mutant by immunoprecipitation with the anti‐GFP antibody followed by immunoblotting with the anti‐Myc antibody in cells transfected with His‐Myc‐ubiquitin and GFP‐KIF11 or its K953R mutant together with control or SYVN1 siRNAs (k), Flag vector or Flag‐SYVN1 plasmids (m), and the relative KIF11 ubiquitination and UFMylation level were determined by densitometry (l, n and o, *n* = 3 independent experiments). Data are presented as mean ± SEM. **p < 0.01, ***p < 0.001, ****p < 0.0001; ns, not significant.

We then sought to investigate the linkage specificity for the ubiquitination of KIF11, by using two ubiquitin mutants. The K48 mutant contains only a single lysine residue, lysine 48, with all of the other lysines mutated to arginines. The K63 mutant contains only a single lysine residue, lysine 63, with all of the other lysines mutated to arginines. It is well established that ubiquitin linkages at K48 predominantly mediate proteasomal degradation of proteins, whereas linkages at K63 are more generally involved in the modulation of protein‐protein interactions. Through the analysis of KIF11 ubiquitination patterns, we observed that the K48 site of ubiquitin was both necessary and sufficient for the polyubiquitin chain assembly on KIF11 in response to UFL1 depletion (**Figure** **5**a). The K48 linkage‐specific ubiquitination of KIF11 was confirmed by examination of the decrease in KIF11 ubiquitination upon UFL1 overexpression (Figure 5b). These results indicate that UFL1‐mediated KIF11 UFMylation impedes the degradation of KIF11 by suppressing its K48 linkage‐specific ubiquitination, thereby enhancing its protein stability.

### 2.5 KIF11 UFMylation Antagonizes Its Ubiquitination by Synoviolin 1 (SYVN1)

To further investigate the interplay between KIF11 UFMylation and ubiquitination, we used the UbiBrowser software to predict the E3 ubiquitin ligase(s) mediating KIF11 ubiquitination. Ten high‐confidence E3 ligases were identified, including SYVN1, tripartite motif 25 (TRIM25), S‐phase kinase‐associated protein 2 (SKP2), fizzy‐related protein 1 (FZR1), neuronally expressed developmentally downregulated 4 (NEDD4), Smad ubiquitination regulatory factor 1 (SMURF1), mind bomb 1 (MIB1), STIP1 homology and U‐Box containing protein 1 (STUB1), casitas B‐lineage lymphoma (CBL), and cell‐division cycle protein 20 homologue (CDC20). By using specific siRNAs targeting these E3 ligases, we found that only knockdown of SYVN1, NEDD4, and CDC20 significantly decreased KIF11 ubiquitination (Figure 5c–h; Figure S4a–g, Supporting Information).

Next, we sought to determine, among SYVN1, NEDD4, and CDC20, which E3(s) mediate KIF11 ubiquitination at the K953 residue to compete with its UFMylation. We examined the effect of depleting each E3 on the ubiquitination of the K953R mutant. Strikingly, the decrease in KIF11 ubiquitination by depleting SYVN1, but not NEDD4 or CDC20, was abolished by the K953R mutation (Figure 5i–l; Figure S5a,b, Supporting Information). Consistent with this observation, SYVN1‐induced KIF11 ubiquitination was completely abolished by the K953R mutation (Figure 5m,n). In addition, KIF11 UFMylation was significantly blocked by SYVN1‐induced KIF11 ubiquitination (Figure 5m,o). Collectively, these results suggest a competition between UFL1‐mediated KIF11 UFMylation and SYVN1‐mediated KIF11 ubiquitination at the K953 residue.

### 2.6 UFMylation of KIF11 Contributes to Its Functions in Photoreceptor Cilium Integrity and Retinal Homeostasis

**Figure 6:**
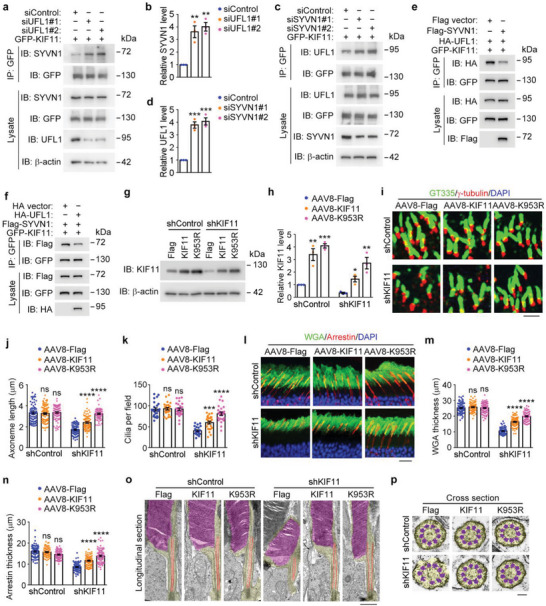
KIF11‐mediated photoreceptor cilium integrity and retinal homeostasis are modulated by its UFMylation. a,b) Analysis of the interaction between KIF11 and SYVN1 by immunoprecipitation with the anti‐GFP antibody followed by immunoblotting with the anti‐SYVN1 antibody in cells transfected with GFP‐KIF11 together with control or UFL1 siRNAs (a). Relative SYVN1 levels were determined by densitometry (b, *n* = 3 independent experiments) c,d) Analysis of the interaction between KIF11 and UFL1 by immunoprecipitation with the anti‐GFP antibody followed by immunoblotting with the anti‐UFL1 antibody in cells transfected with GFP‐KIF11 together with control or SYVN1 siRNAs (c). Relative UFL1 levels were determined by densitometry (d, *n* = 3 independent experiments). e) Analysis of the interaction between KIF11 and UFL1 by immunoprecipitation with the anti‐GFP antibody followed by immunoblotting with the anti‐HA antibody in cells transfected with GFP‐KIF11, HA‐UFL1, and Flag vector or Flag‐SYVN1 plasmids. f) Analysis of the interaction between KIF11 and SYVN1 by immunoprecipitation with the anti‐GFP antibody followed by immunoblotting with the anti‐Flag antibody in cells transfected with GFP‐KIF11, Flag‐SYVN1, and HA vector or HA‐UFL1 plasmids. g,h) Immunoblot analysis of KIF11 and β‐actin in retinas injected with control or KIF11‐shRNA adenoviruses, together with AAV‐Flag, AAV‐KIF11, or AAV‐KIF11‐K953R (g), and the relative KIF11 level were determined by densitometry (h, *n* = 3 independent experiments). i–k) Immunofluorescence images (i) and quantification of ciliary axoneme length (j, *n* = 80 fields from 12 mice of three independent experiments) and ciliary density (m, *n* = 24 eyes from 12 mice of three independent experiments) in retinas injected with control or KIF11‐shRNA adenoviruses, together with AAV‐Flag, AAV‐KIF11, or AAV‐KIF11‐K953R. Scale bar, 3 µm. l–n) Immunofluorescence images (l) and quantification of membranous disc thickness in rods (m) and cones (n) in retinas injected with control or KIF11‐shRNA adenovirus, together with AAV‐Flag, AAV‐KIF11, or AAV‐KIF11‐K953R (m and n, *n* = 60 fields from 12 mice of three independent experiments). Scale bar, 20 µm. o,p) Transmission electron microscopy images of longitudinal sections of photoreceptors (o) and cross sections of photoreceptor ciliary axonemes (p) in retinas injected with control or KIF11‐shRNA adenoviruses, together with AAV‐Flag, AAV‐KIF11, or AAV‐KIF11‐K953R. Scale bars, 1 µm (o) and 0.1 µm (p). Data are presented as mean ± SEM. *p < 0.5, **p < 0.01, ***p < 0.001, ****p < 0.0001; ns, not significant.

To understand the interplay between KIF11 UFMylation and ubiquitination, we examined whether UFL1 competes with SYVN1 for binding to KIF11. Indeed, we found that siRNA‐mediated depletion of UFL1 promoted the interaction between KIF11 and SYVN1 (**Figure** **6**a,b). Depletion of SYVN1 also enhanced the interaction of UFL1 with KIF11 (Figure 6c,d). In addition, overexpression experiments confirmed the competition between UFL1 and SYVN1 for binding to KIF11 (Figure 6e,f). Taken together, these findings suggest that UFL1 competes with SYVN1 for binding to KIF11 and subsequently increases KIF11 UFMylation and reduces its ubiquitination, thereby impeding its proteasomal degradation and enhancing its protein stability.

We then sought to examine the role of KIF11 UFMylation in photoreceptor cilium integrity. KIF11‐depleted mice were intravitreally injected with adeno‐associated viruses (AAVs) encoding KIF11 wild‐type or its K953R mutant. Immunoblotting revealed that the level of KIF11 K953R in the retina was higher than that of the wild‐type (Figure 6g,h), confirming that this mutation enhances KIF11 stability. In addition, immunofluorescence microscopy revealed that the K953R mutant was more effective than the wild‐type in rescuing the KIF11 depletion‐induced defects in photoreceptor cilia (Figure 6i–k) and membranous discs (Figure 6l–n). Consistent with these results, transmission electron microscopy of longitudinal and cross sections of photoreceptor membranous discs and ciliary axonemes revealed that the K953R mutant of KIF11 had a stronger effect than the wild‐type in rescuing the photoreceptor ultrastructure (Figure 6o,p; Figure S6a–c, Supporting information).

Next, we investigated the impact of KIF11 UFMylation on retinal homeostasis. Histopathological analysis of retinal morphology revealed that the K953R mutant was more effective than wild‐type KIF11 in rescuing KIF11 depletion‐induced decrease in the ONL thickness (Figure S6d,e, Supporting Information). Additionally, fundus photography revealed that the retinal DLDs induced by KIF11 depletion were more effectively eliminated by the K953R mutant compared to wild‐type KIF11 (Figure S6f,g, Supporting Information). Collectively, these results suggest that UFMylation plays a crucial role in the maintenance of photoreceptor cilium integrity and retinal homeostasis by KIF11.

## 3 Discussion

Photoreceptors detect light by concentrating the light‐sensing opsins and the downstream phototransduction machinery in the modified primary cilium filled with stacked membrane discs. This specialized cilium is a remarkably dynamic organelle that turns over its contents to replenish photodamaged opsins. During the process of renewal, the discrete packages of old discs are shed and engulfed by the retinal pigment epithelium. Meanwhile, the new discs are continuously formed and push older discs toward the tip. Recent studies have indicated that ciliary shedding contributes to both disc shedding and biogenesis.^[^ ^6^, ^28^ ^]^ The findings of our present work demonstrate that KIF11 depletion triggers a reduction in the length of both ciliary axonemes and membranous discs, indicating that the mechanistic underpinnings of disc shedding may be attributable to the ciliary shedding process.

KIF11 is a kinesin family member that primarily functions in driving centrosome separation and bipolar spindle formation during mitosis.^[^ ^13^ ^]^ However, there is increasing evidence that KIF11 is crucial in non‐mitotic processes. For example, KIF11 has been demonstrated to have microtubule polymerase activity and regulate axon growth.^[^ ^29^, ^30^ ^]^ KIF11 has also been reported to control centrosome migration and vesicle transport in post‐mitotic cells.^[^ ^31^, ^32^ ^]^ In addition, our previous work and other studies have shown an enrichment of KIF11 in the basal body to promote cilium formation.^[^ ^14^, ^15^, ^33^ ^]^ Our current work demonstrates that KIF11 localizes to the photoreceptor connecting cilium and daughter centriole and plays a critical role in the maintenance of photoreceptor cilium integrity and retinal homeostasis, furthering our understanding of the importance of KIF11 in non‐mitotic processes. Nevertheless, the precise molecular mechanisms underlying KIF11‐mediated cilium formation have yet to be elucidated. Recent studies have indicated that defects in ciliary transition zone impair the formation and biological activity of ciliary ectosomes, which are extracellular vesicles released from the cilium into the extracellular space, contributing to the process of ciliary shedding.^[^ ^34^ ^]^ Our present study demonstrates that KIF11 localizes to the photoreceptor connecting cilium, which is the equivalent of a canonical ciliary transition zone. These findings imply that KIF11 might be involved in the process of ciliary shedding through regulating the formation of ciliary ectosomes.

**Figure 7:**
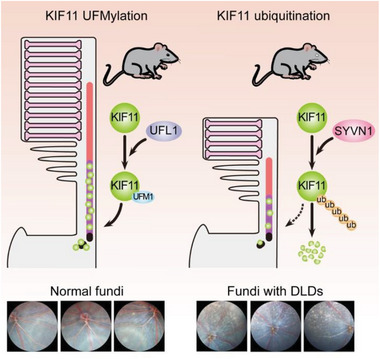
A scheme illustrating the interplay between KIF11 UFMylation and ubiquitination in the maintenance of photoreceptor cilium integrity and retinal homeostasis.

Moreover, *KIF11* gene mutations have been identified in patients with various retinal diseases including MLCRD (microcephaly, lymphedema, and chorioretinal dysplasia), CDMMR (chorioretinal dysplasia, microcephaly, and mental retardation), and FEVR (familial exudative vitreoretinopathy).^[^ ^35^, ^36^ ^]^ These accumulating results, together with our finding that the KIF11 is required for the maintenance of photoreceptor cilium integrity and retinal homeostasis (**Figure** **7**), indicate that this kinesin might serve as a marker for the diagnosis of retinal diseases. Notably, genetic variants in *KIF11* have also been identified in patients with type 2 diabetes and diabetic retinopathy,^[^ ^37^, ^38^ ^]^ leading us to hypothesize that KIF11 may also play a pivotal role in the pathogenesis of diabetic retinopathy. This connection warrants further exploration, as understanding the involvement of KIF11 in such common and debilitating conditions could reveal notable insights into the underpinnings of retinal diseases and open new avenues for diagnostic and therapeutic strategies.

KIF11 is known to undergo post‐translational modifications including phosphorylation, acetylation, and ubiquitination. For example, KIF11 can be phosphorylated at threonine 926 and serine 1033 by cyclin‐dependent kinase 1 and Nek6, respectively, and the phosphorylation is crucial for the formation of bipolar spindles.^[^ ^39^, ^40^, ^41^, ^42^ ^]^ The motor activity of KIF11 has been demonstrated to be significantly enhanced by its acetylation at lysine 146.^[^ ^43^ ^]^ In addition, the ubiquitination of KIF11 at lysine 891 and lysine 899 by the E3 ligase FBXO30 has been shown to regulate KIF11 protein level to mediate centrosome homeostasis and genome stability.^[^ ^44^ ^]^ However, there is relatively limited information about the interplay of different modifications of KIF11. Our finding that KIF11 undergoes mono‐UFMylation at lysine 953, which increases KIF11 stability by antagonizing its ubiquitination, thus adds a further level of complexity to the post‐translational control of KIF11.

UFM1 is a ubiquitin‐like protein that can be conjugated to its substrates via an E1‐E2‐E3 enzymatic cascade.^[^ ^26^, ^45^ ^]^ UFMylation participates in various biological processes including endoplasmic reticulum stress, oxidative stress, DNA damage response, hematopoiesis, fatty acid metabolism, G‐protein‐coupled receptor biogenesis, and neurodevelopment.^[^ ^27^, ^46^, ^47^, ^48^, ^49^, ^50^, ^51^, ^52^, ^53^, ^54^, ^55^, ^56^ ^]^ Deficiency in UFMylation leads to embryonic lethality in mice and has been implicated in a variety of human diseases.^[^ ^46^ ^]^ However, only a limited number of proteins have been identified to date as the physiological substrates for UFMylation.^[^ ^45^ ^]^ In the present study, our data demonstrate that KIF11 is a novel substrate of UFMylation and reveal a critical role for KIF11 UFMylation in maintaining photoreceptor cilium integrity and retinal homeostasis. Studies have demonstrated that oxidative stress and DNA damage are involved in the pathogenesis of various retinal diseases, such as retinopathy of prematurity, macular degeneration, and diabetic retinopathy. These findings suggest that the observed reduction in KIF11 UFMylation may be intricately linked to oxidative stress or DNA damage associated with retinal diseases. Furthermore, it will be critically important to continue investigations using clinical samples to explore the involvement of KIF11 UFMylation in retinal diseases.

Enormous efforts have been made over the past decades to develop therapeutics for retinal diseases. For example, gene therapy using lentiviruses or AAVs is currently considered a promising approach to prevent or treat retinal diseases.^[^ ^57^ ^]^ The first clinical trial using AAVs was initiated in patients with Leber congenital amaurosis caused by mutations in the retinal pigment epithelium 65 (*RPE65*) gene, with the method now approved by the Food and Drug Administration and representing one of the very first successes of gene therapy.^[^ ^58^, ^59^ ^]^ In addition, gene mutations that cause retinal diseases might be efficiently corrected by genome editing technologies, which can now permanently and precisely replace or remove the mutations that cause retinal diseases, at least for retinitis pigmentosa and Leber congenital amaurosis in in vivo models.^[^ ^60^, ^61^ ^]^ In the present study, we demonstrate that KIF11 depletion‐induced photoreceptor and retinal deficits are efficiently ameliorated by intravitreal injection of KIF11 wild‐type or K953R mutant AAVs. Given that *KIF11* gene mutations have been identified in several retinal diseases, it is tempting to speculate that gene therapy might be a promising strategy for the treatment of KIF11‐associated retinal diseases.

## 4 Experimental Section

### Animal Care and Use

All animal experiments were performed according to protocols approved by the Animal Care and Use Committee of Shandong Normal University (AEECSDNU2021048). Mice were housed in a temperature‐controlled facility with a 12 hour‐light/12 hour‐dark cycle, specific pathogen‐free, in individual cages and provided food and water.

### Intravitreal Injection of Adenoviruses

Adenoviruses targeting the mouse *KIF11* gene were generated using the pCMV‐MCS vector, and adeno‐associated viruses encoding KIF11 were generated using the pDC315‐Flag vector (GeneChem, Shanghai, China) as previously described.^[^ ^12^, ^18^ ^]^ The sequences targeting KIF11 were 5′‐TATTGTCTTCAGGTCTTCA‐3′ and 5′‐ TGCAGGTCAGATTTACACT‐3′, and the negative control sequence was 5′‐TTCTCCGAACGTGTCACGT‐3′. For intravitreal injection, 8‐week‐old C57BL/6J mice were anesthetized with 2% isoflurane and 0.5% oxybuprocaine hydrochloride (Santen Pharmaceuticals) applied to the corneal surface. The iris was dilated using 0.5% tropicamide phenylephrine (Santen), and mice were intravitreally injected with 1 µL solution containing 1.2 × 10^12^ PFU mL^−1^ KIF11‐shRNA adenoviruses using a 34‐gauge Hamilton needle and syringe as described previously^[^ ^12^ ^]^ (Hamilton Company, Reno, NV). After the injection, 0.5% proparacaine and TobraDex (Alcon, Geneva, Switzerland) were applied to the eye to minimize pain and reduce the risk of inflammation.

### Fundus Photography

Mice were anesthetized by intraperitoneal injection of 2.5% tribromoethanol (Sigma‐Aldrich, St. Louis, MO). The body temperature of mice was monitored at 37.0 ± 0.5 °C (TC‐100, Eaton). Each pupil was dilated with 0.5% tropicamide phenylephrine (Santen), and ophthalmic gel (Gonak; Akorn, Lake Forest, IL) was then applied to the eye. Retinal images were captured using an OPTO‐RIS retinal imaging system (Optoprobe Science, Burnaby, Canada), with a recognizable anatomical point, two optic‐disc diameter from the optic disc, for each mouse.

### Histopathological Analysis

Eye samples were fixed in 4% paraformaldehyde overnight at 4 °C. Then, the cornea and lens were removed and the retinas were fixed for an additional 2 h at room temperature. Retinas were embedded in paraffin, and the 4‐µm retinal sections were prepared. Sections encompassing the optic nerve were selected for H&E staining using the standard method. For retinal thickness measurement, sections from the same eccentricity and eye cups embedded in the same orientation were selected. Images were observed and photographed with a DM3000 microscope (Leica, Wezlar, Germany).

### Immunofluorescence Staining

To stain membranous discs, mice were euthanized with an overdose of pentobarbital sodium. Mice were then transcardially perfused with a fixative solution containing 4% paraformaldehyde with 80 mm PIPES (pH 6.8), 5 mm EGTA, and 2 mm MgCl_2_. The eye samples were quickly removed and post‐fixed in the same fixative solution overnight at 4 °C. After removing the cornea and lens, eye cups were embedded in 5% low‐melt agarose (A600015‐0025; Sangon Biotech, Shanghai, China) and cut with a vibratome (VT1200S; Leica) into 80‐µm slices. Retinal sections were blocked in a solution containing 7% goat serum in 0.5% Triton X‐100 for 1 h at room temperature. Then, slices were incubated with Alexa Fluor 488‐conjugated WGA (1 µg mL^−1^; W11261; Thermo Fisher Scientific, Waltham, MA) for 45 min at room temperature and washed three times in phosphate‐buffered saline (PBS). Sections were incubated with the anti‐arrestin antibody (MAB15282, Millipore) at 4 °C overnight, followed by staining with Alexa Fluor 568‐conjugated secondary antibodies (A10042; Thermo Fisher Scientific) at room temperature for 1 h. Slides were washed in PBS three times and stained with DAPI. After sections were washed another three times in PBS and mounted onto slides in glycerol, images were taken using the Dragonfly 200 confocal imaging system (Andor Technology, Belfast, UK).

To stain photoreceptor cilia, eye samples were fixed in 4% paraformaldehyde for 30 s at room temperature, cryo‐embedded in Tissue‐Tek OCT (Sakura), and 10‐µm frozen sections were prepared using a freezing microtome (CM3050S; Leica). Frozen sections were incubated with antibodies against polyglutamylated tubulin (GT335, AG‐20B‐0020, AdipoGen Life Sciences, San Diego, CA), Arl13b (17711‐1‐AP; Proteintech), or KIF11 (HP1006916, Sigma‐Aldrich) at 4 °C overnight and then washed three times in PBS. Slides were then incubated with Alexa Fluor 568‐conjugated or Alexa Fluor 488‐conjugated (A21202; Thermo Fisher Scientific) secondary antibodies for 1 h at room temperature. After washing in PBS three times, slides were stained with DAPI. Images were taken using a Leica SP8 confocal microscope and analyzed with the 3D‐analysis tool in Leica Application Suite X software.

### Transmission Electron Microscopy

Eyeballs were fixed with 0.25% glutaraldehyde in 0.1 m sodium cacodylate for 4 h at room temperature. After removal of the lens and cornea, eye cups were fixed in the same fixative solution at 4 °C overnight followed by post‐fixation in 1% osmium tetroxide for 1 h at room temperature. Retinal samples were then dehydrated through a series of ethanol concentrations, infiltrated with resin, embedded in Spurr low viscosity resin, and cured for three days at 65 °C. Finally, 50‐nm ultrathin sections were prepared and stained with uranyl acetate and lead citrate as described previously.^[^ ^62^ ^]^ Images were taken with an HT‐7800 transmission electron microscope (Hitachi, Tokyo, Japan) at 80 kV.

### Cells, Plasmids, and siRNAs

HEK293T and RPE1 cells were purchased from the American Type Culture Collection and maintained in Dulbecco's modified Eagle medium (DMEM, Biological Industries, Haemek, Israel) and DMEM:F12 (1:1) medium with 10% fetal bovine serum (FBS; HyClone, Cytiva, Marlborough, MA), respectively. Plasmids for UFM1 and UFL1 were described previously.^[^ ^52^ ^]^ The KIF11 cDNA and the deletion mutant cDNAs were cloned into the pEGFP‐C1 vector. The lysine to arginine mutations in KIF11 were generated by PCR and site‐directed mutagenesis. The SYVN1 cDNA was cloned into the pcDNA3.1‐FLAG vector. The His‐Myc‐ubiquitin plasmid was described previously.^[^ ^62^ ^]^ The sequences of siRNAs used in this study were as follows: siUFL1#1, 5′‐GGAACUUGUUAAUAGCGGA‐3′; siUFL1#2, 5′‐GAGGAGUAAUUUUUACGGA‐3′; siKIF11#1, 5′‐CUGAAGACCUGAAGACAAU‐3′; and siKIF11#2, 5′‐CAACAAGGAUGAAGUCUAU‐3′. For plasmid transfections, HEK293T cells were seeded in plates without antibiotics. After cell adherence, the transfection mixture containing plasmids and polyethyleneimine (Polysciences, Warrington, PA) was added into the culture medium. The medium was changed to fresh DMEM with 10% FBS 12 h after transfection. siRNA transfections were performed with Lipofectamine RNAiMAX (Invitrogen, Waltham, MA), according to the manufacturer's instructions.

### Detection of KIF11 UFMylation and Ubiquitination

To detect KIF11 UFMylation, after transfection of the appropriate constructs for 36 h, cells were lysed by boiling in a buffer containing 150 mm Tris‐HCl (pH 8.0), 5% sodium dodecyl sulfate (SDS) and 30% glycerol for 10 min. Cell lysates were then diluted 20‐fold in a solution containing 50 mm Tris‐HCl (pH 8.0), 150 mm NaCl, 0.5% NP‐40, and 2 mm N‐ethylmaleimide, and the protease inhibitor cocktail (Roche, Basel, Switzerland). After incubation with anti‐GFP antibody‐conjugated agaroses (D153‐8, MBL Life Science, Woburn, MA) at 4 °C overnight, the bound proteins were subjected to SDS‐PAGE and immunoblotting. For the detection of endogenous KIF11 UFMylation, whole retinal lysates were incubated with agarose beads (20 421, Thermo Fisher Scientific) coated with the anti‐KIF11 antibody (HP1006916, Sigma‐Aldrich), anti‐UFM1 antibody (ab109305, Abcam, Cambridge, UK), or the rabbit IgG (66467‐1‐Ig, Proteintech, Rosemont, IL). The immunoprecipitates were then subjected to SDS‐PAGE and immunoblotting.

To detect KIF11 ubiquitination, cells were transfected with the appropriate constructs for 24 h followed by treatment with 10 µm MG132 for another 10 h. Then, cells were lysed by boiling in a buffer containing 1% NP‐40, 0.5% sodium deoxycholate, 1% SDS, 10 mm N‐ethylmaleimide, 1 mm phenylmethylsulfonyl fluoride, 1 mm NaV_3_O_4_, and 1 mm NaF for 10 min and diluted ten‐fold with a lysis buffer containing 50 mm Tris (pH 7.5), 150 mm NaCl, 1 mm EDTA, 3% glycerin, 1% NP40, and the protease inhibitor cocktail (Roche). After incubation with anti‐GFP antibody‐conjugated agarose at 4 °C overnight, the immunoprecipitates were subjected to SDS‐PAGE and immunoblotting.

### Immunoblotting of Retinal Samples

After removal of the cornea and lens, retinas were lysed in a cold lysis buffer containing 50 mm Tris (pH 7.5), 150 mm NaCl, 1 mm EDTA, 3% glycerin, 1% NP40, and the protease inhibitor cocktail (Roche) using a tissue grinding machine (JXFSTPRP‐24L; Jingxin, Shanghai, China). Equal amounts of total retinal proteins (30–40 µg) were resolved by SDS‐PAGE and transferred onto polyvinylidene difluoride membranes (MilliporeSigma, Burlington, MA) and subjected to immunoblotting, using anti‐KIF11 (HP1006916, Sigma‐Aldrich), anti‐UFL1 (HPA030559, Sigma‐Aldrich), anti‐UFM1 (ab109305, Abcam, Cambridge, UK), or anti‐β‐actin (A5316, Sigma‐Aldrich) antibodies.

### GST Pull‐Down

GST‐UFL1 proteins were expressed in *E. coli* and purified with glutathione resins (L00206, GenScript) as described previously.^[^ ^52^ ^]^ For in vitro binding assays, 100 ng of purified GST or GST‐UFL1 was incubated with glutathione resins for 4 h at 4 °C. Beads were washed three times with the lysis buffer and incubated with 100 ng of Myc‐KIF11 protein (TP318842, OriGene) overnight at 4 °C with gentle rotation. The bound proteins were eluted with the loading buffer and analyzed by immunoblotting with the anti‐GST antibody (AB0003, Abways).

### Mass Spectrometry

To identify KIF11‐interacting proteins, the whole retinal lysate was immunoprecipitated by incubation with agarose beads and the anti‐KIF11 antibody at 4 °C overnight. The immunoprecipitate was resolved by SDS‐PAGE and subjected to mass spectrometry on a Q‐Exactive HF Mass Spectrometer (Thermo Fisher Scientific). Mass spectrometric analysis was performed using Proteome Discoverer 2.4 by PTM Biolabs (Hangzhou, China).

### Quantitative RT‐PCR

Total RNAs were isolated from retinal samples or cells using the TRIzol reagent (Invitrogen) according to the manufacturer's protocol, followed by cDNA synthesis using M‐MLV reverse transcriptase (Promega, Madison, WI). Quantitative real‐time PCR was then performed in triplicates using a 7500 HT Sequence Detection System and the Power SYBR Green PCR Master Mix Kit (Applied Biosystems, Waltham, MA). All reactions were performed with GAPDH as control, and the KIF11 mRNA level was normalized to the GAPDH mRNA level. The following primers were used: mouse KIF11, 5′‐CAACCACCAATGATGCTAAACAG‐3′ and 5′‐GAGCCTCCCTCTCTTCATCCA‐3′; mouse GAPDH, 5′‐ATGAGGTCCACCACCCTGTT‐3′ and 5′‐ATCACTGCCACCCAGAAGAC‐3′; human KIF11, 5′‐GAACAATCA TTAGCAGCAGAA‐3′ and 5′‐TCAGTATAGACACCA CAGTTG‐3′; and human GAPDH, 5′‐GGAGCGAGATCCCTCCAAAAT‐3′ and 5′‐GGCTGTTGTCATACTTCTCATGG‐3′.

### Statistical Analysis

The GraphPad Prism 8.0 software (GraphPad Software, La Jolla, CA) was used for statistical analysis. Experimental data were presented as mean ± SEM. For normally distributed data, Student's *t*‐test was performed to evaluate differences between two groups, and two‐way analysis of variance (ANOVA) was employed to compare three or more groups. For data not normally distributed, non‐parametric analyses were performed. A p‐value < 0.05 was considered statistically significant.

## Conflict of Interest

The authors declare no conflict of interest.

## Supporting information

Supporting Information

Supplemental Table 1

## Data Availability

The data that support the findings of this study are available from the corresponding author upon reasonable request.

## References

[1] J. B. Lin , R. Narayanan , E. Philippakis , Y. Yonekawa , R. S. Apte , Nat. Rev. Dis. Primers 2024, 10, 18. 38485969 10.1038/s41572-024-00501-5

[2] D. A. Antonetti , P. S. Silva , A. W. Stitt , Nat. Rev. Endocrinol. 2021, 17, 195. 33469209 10.1038/s41574-020-00451-4 PMC9053333

[3] D. Tonade , T. S. Kern , Prog. Retinal Eye Res. 2021, 83, 100919. 10.1016/j.preteyeres.2020.100919 PMC8113320 33188897

[4] R. S. Apte , N. Engl. J. Med. 2021, 385, 539. 34347954 10.1056/NEJMcp2102061 PMC9369215

[5] S. Temple , Cell Stem Cell 2023, 30, 512. 37084729 10.1016/j.stem.2023.03.017 PMC10201979

[6] W. J. Spencer , T. R. Lewis , J. N. Pearring , V. Y. Arshavsky , Trends Cell Biol. 2020, 30, 904. 32900570 10.1016/j.tcb.2020.08.005 PMC7584774

[7] T. Baden , T. Euler , P. Berens , Nat. Rev. Neurosci. 2020, 21, 5. 31780820 10.1038/s41583-019-0242-1

[8] W. B. Thoreson , D. M. Dacey , Physiol. Rev. 2019, 99, 1527. 31140374 10.1152/physrev.00027.2018 PMC6689740

[9] W. Baehr , C. Hanke‐Gogokhia , A. Sharif , M. Reed , T. Dahl , J. M. Frederick , G. Ying , Prog. Retinal Eye Res. 2019, 71, 26. 10.1016/j.preteyeres.2018.12.004 PMC6707082 30590118

[10] K. M. Bujakowska , Q. Liu , E. A. Pierce , Cold Spring Harb Perspect Biol. 2017, 9, a028274. 28289063 10.1101/cshperspect.a028274 PMC5629997

[11] J. Ran , J. Zhou , Acta Pharmacol. Sin. 2020, 41, 1410. 32753732 10.1038/s41401-020-0486-3 PMC7656575

[12] J. Ran , Y. Zhang , S. Zhang , H. Li , L. Zhang , Q. Li , J. Qin , D. Li , L. Sun , S. Xie , X. Zhang , L. Liu , M. Liu , J. Zhou , Adv. Sci. 2022, 9, 2105365. 10.1002/advs.202105365 PMC9313505 35619548

[13] B. J. Mann , P. Wadsworth , Trends Cell Biol. 2019, 29, 66. 30220581 10.1016/j.tcb.2018.08.004

[14] J. Ran , H. Li , Y. Zhang , F. Yu , Y. Yang , C. Nie , S. Yang , D. Li , J. Zhou , M. Liu , Sci. Bull. 2021, 66, 1620. 10.1016/j.scib.2021.02.001 36654295

[15] A. A. Zalenski , S. Majumder , K. De , M. Venere , Sci. Rep. 2020, 10, 13946. 32811879 10.1038/s41598-020-70787-4 PMC7434902

[16] J. M. Robitaille , R. M. Gillett , M. A. LeBlanc , D. Gaston , M. Nightingale , M. P. Mackley , S. Parkash , J. Hathaway , A. Thomas , A. Ells , E. I. Traboulsi , E. Héon , M. Roy , S. Shalev , C. V. Fernandez , C. MacGillivray , K. Wallace , S. Fahiminiya , J. Majewski , C. R. McMaster , K. Bedard , JAMA Ophthalmol. 2014, 132, 1393. 25124931 10.1001/jamaophthalmol.2014.2814

[17] H. Hu , X. Xiao , S. Li , X. Jia , X. Guo , Q. Zhang , Br. J. Ophthalmol. 2016, 100, 278. 26472404 10.1136/bjophthalmol-2015-306878

[18] D. J. Von Seggern , E. Aguilar , K. Kinder , S. K. Fleck , J. C. Gonzalez Armas , S. C. Stevenson , P. Ghazal , G. R. Nemerow , M. Friedlander , Mol. Ther. 2003, 7, 27. 12573615 10.1016/s1525-0016(02)00030-8

[19] S. Schmitz‐Valckenberg , M. Pfau , M. Fleckenstein , G. Staurenghi , J. R. Sparrow , A. Bindewald‐Wittich , R. F. Spaide , S. Wolf , S. R. Sadda , F. G. Holz , Prog. Retinal Eye Res. 2021, 81, 100893. 10.1016/j.preteyeres.2020.100893 PMC12906268 32758681

[20] K. Million , J. Larcher , J. Laoukili , D. Bourguignon , F. Marano , F. Tournier , J. Cell Sci. 1999, 112, 4357. 10564653 10.1242/jcs.112.23.4357

[21] U. Wolfrum , Cell Motil. Cytoskeleton 1995, 32, 55. 8674134 10.1002/cm.970320107

[22] C. D. Bridges , Invest. Ophthalmol. Vis. Sci. 1981, 20, 8. 6969714

[23] V. V. Gurevich , J. L. Benovic , J. Biol. Chem. 1995, 270, 6010. 7890732 10.1074/jbc.270.11.6010

[24] T. Caspary , C. E. Larkins , K. V. Anderson , Dev. Cell 2007, 12, 767. 17488627 10.1016/j.devcel.2007.03.004

[25] K. Tatsumi , Y. S. Sou , N. Tada , E. Nakamura , S. Iemura , T. Natsume , S. H. Kang , C. H. Chung , M. Kasahara , E. Kominami , M. Yamamoto , K. Tanaka , M. Komatsu , J. Biol. Chem. 2010, 285, 5417. 20018847 10.1074/jbc.M109.036814 PMC2820770

[26] M. Komatsu , T. Chiba , K. Tatsumi , S. Iemura , I. Tanida , N. Okazaki , T. Ueno , E. Kominami , T. Natsume , K. Tanaka , EMBO J. 2004, 23, 1977. 15071506 10.1038/sj.emboj.7600205 PMC404325

[27] H. M. Yoo , S. H. Kang , J. Y. Kim , J. E. Lee , M. W. Seong , S. W. Lee , S. H. Ka , Y. S. Sou , M. Komatsu , K. Tanaka , S. T. Lee , D. Y. Noh , S. H. Baek , Y. J. Jeon , C. H. Chung , Mol. Cell 2014, 56, 261. 25219498 10.1016/j.molcel.2014.08.007

[28] H. Zhao , Q. Li , J. Zhou , Sci. Bull. 2023, 68, 2674. 10.1016/j.scib.2023.09.027 37833188

[29] Y. Chen , W. O. Hancock , Nat. Commun. 2015, 6, 8160. 26437877 10.1038/ncomms9160 PMC4600729

[30] K. A. Myers , P. W. Baas , J. Cell Biol. 2007, 178, 1081. 17846176 10.1083/jcb.200702074 PMC2064629

[31] Y. Wakana , J. Villeneuve , J. van Galen , D. Cruz‐Garcia , M. Tagaya , V. Malhotra , J. Cell Biol. 2013, 202, 241. 23857769 10.1083/jcb.201303163 PMC3718972

[32] C. M. Whitehead , J. B. Rattner , J. Cell Sci. 1998, 111, 2551. 9701554 10.1242/jcs.111.17.2551

[33] L. Li , J. Ran , Cell Death Dis. 2024, 15, 47. 38218748 10.1038/s41419-024-06428-9 PMC10787775

[34] L. Wang , X. Wen , Z. Wang , Z. Lin , C. Li , H. Zhou , H. Yu , Y. Li , Y. Cheng , Y. Chen , G. Lou , J. Pan , M. Cao , Nat. Commun. 2022, 13, 3997. 35810181 10.1038/s41467-022-31751-0 PMC9271036

[35] P. Ostergaard , M. A. Simpson , A. Mendola , P. Vasudevan , F. C. Connell , A. van Impel , A. T. Moore , B. L. Loeys , A. Ghalamkarpour , A. Onoufriadis , I. Martinez‐Corral , S. Devery , J. G. Leroy , L. van Laer , A. Singer , M. G. Bialer , M. McEntagart , O. Quarrell , G. Brice , R. C. Trembath , S. Schulte‐Merker , T. Makinen , M. Vikkula , P. S. Mortimer , S. Mansour , S. Jeffery , Am. J. Hum. Genet. 2012, 90, 356. 22284827 10.1016/j.ajhg.2011.12.018 PMC3276660

[36] J. M. Robitaille , R. M. Gillett , M. A. LeBlanc , D. Gaston , M. Nightingale , M. P. Mackley , S. Parkash , J. Hathaway , A. Thomas , A. Ells , E. I. Traboulsi , E. Heon , M. Roy , S. Shalev , C. V. Fernandez , C. MacGillivray , K. Wallace , S. Fahiminiya , J. Majewski , C. R. McMaster , K. Bedard , JAMA Ophthalmol. 2014, 132, 1393. 25124931 10.1001/jamaophthalmol.2014.2814

[37] R. Sladek , G. Rocheleau , J. Rung , C. Dina , L. Shen , D. Serre , P. Boutin , D. Vincent , A. Belisle , S. Hadjadj , B. Balkau , B. Heude , G. Charpentier , T. J. Hudson , A. Montpetit , A. V. Pshezhetsky , M. Prentki , B. I. Posner , D. J. Balding , D. Meyre , C. Polychronakos , P. Froguel , Nature 2007, 445, 881. 17293876 10.1038/nature05616

[38] D. Peng , J. Wang , R. Zhang , F. Jiang , C. H. T. Tam , G. Jiang , T. Wang , M. Chen , J. Yan , S. Wang , D. Yan , Z. He , R. C. W. Ma , Y. Bao , C. Hu , W. Jia , Sci. Rep. 2017, 7, 8812. 28821857 10.1038/s41598-017-09010-w PMC5562862

[39] A. Blangy , L. Arnaud , E. A. Nigg , J. Biol. Chem. 1997, 272, 19418. 9235942 10.1074/jbc.272.31.19418

[40] A. Blangy , H. A. Lane , P. d'Herin , M. Harper , M. Kress , E. A. Nigg , Cell 1995, 83, 1159. 8548803 10.1016/0092-8674(95)90142-6

[41] J. Rapley , M. Nicolas , A. Groen , L. Regue , M. T. Bertran , C. Caelles , J. Avruch , J. Roig , J. Cell Sci. 2008, 121, 3912. 19001501 10.1242/jcs.035360 PMC4066659

[42] M. T. Bertran , S. Sdelci , L. Regue , J. Avruch , C. Caelles , J. Roig , EMBO J. 2011, 30, 2634. 21642957 10.1038/emboj.2011.179 PMC3155310

[43] J. M. Muretta , B. J. N. Reddy , G. Scarabelli , A. F. Thompson , S. Jariwala , J. Major , M. Venere , J. N. Rich , B. Willard , D. D. Thomas , J. Stumpff , B. J. Grant , S. P. Gross , S. S. Rosenfeld , Proc. Natl. Acad. Sci. USA 2018, 115, E1779. 29432173 10.1073/pnas.1718290115 PMC5828613

[44] Y. Liu , Y. Wang , Z. Du , X. Yan , P. Zheng , Y. Liu , Cell Rep. 2016, 15, 1111. 27117404 10.1016/j.celrep.2016.03.083 PMC4856546

[45] Y. Gerakis , M. Quintero , H. Li , C. Hetz , Trends Cell Biol. 2019, 29, 974. 31703843 10.1016/j.tcb.2019.09.005 PMC6917045

[46] K. Tatsumi , H. Yamamoto‐Mukai , R. Shimizu , S. Waguri , Y. S. Sou , A. Sakamoto , C. Taya , H. Shitara , T. Hara , C. H. Chung , K. Tanaka , M. Yamamoto , M. Komatsu , Nat. Commun. 2011, 2, 181. 21304510 10.1038/ncomms1182 PMC3105337

[47] M. Zhang , X. Zhu , Y. Zhang , Y. Cai , J. Chen , S. Sivaprakasam , A. Gurav , W. Pi , L. Makala , J. Wu , B. Pace , D. Tuan‐Lo , V. Ganapathy , N. Singh , H. Li , Cell Death Differ. 2015, 22, 1922. 25952549 10.1038/cdd.2015.51 PMC4816109

[48] A. T. Egunsola , Y. Bae , M. M. Jiang , D. S. Liu , Y. Chen‐Evenson , T. Bertin , S. Chen , J. T. Lu , L. Nevarez , N. Magal , A. Raas‐Rothschild , E. C. Swindell , D. H. Cohn , R. A. Gibbs , P. M. Campeau , M. Shohat , B. H. Lee , J. Clin. Invest. 2017, 127, 1475. 28263186 10.1172/JCI90193 PMC5373861

[49] Z. Wang , Y. Gong , B. Peng , R. Shi , D. Fan , H. Zhao , M. Zhu , H. Zhang , Z. Lou , J. Zhou , W. G. Zhu , Y. S. Cong , X. Xu , Nucleic Acids Res. 2019, 47, 4124. 30783677 10.1093/nar/gkz110 PMC6486557

[50] X. Hu , H. Zhang , Y. Song , L. Zhuang , Q. Yang , M. Pan , F. Chen , Biosci. Rep. 2020, 40, BSR20191672. 31829413 10.1042/BSR20191672 PMC6944655

[51] C. Chen , E. Itakura , K. P. Weber , R. S. Hegde , M. de Bono , PLoS Genet. 2014, 10, 1004082. 10.1371/journal.pgen.1004082 PMC3945108 24603482

[52] J. Liu , D. Guan , M. Dong , J. Yang , H. Wei , Q. Liang , L. Song , L. Xu , J. Bai , C. Liu , J. Mao , Q. Zhang , J. Zhou , X. Wu , M. Wang , Y. S. Cong , Nat. Cell Biol. 2020, 22, 1056. 32807901 10.1038/s41556-020-0559-z

[53] J. R. Liang , E. Lingeman , T. Luong , S. Ahmed , M. Muhar , T. Nguyen , J. A. Olzmann , J. E. Corn , Cell 2020, 180, 1160. 32160526 10.1016/j.cell.2020.02.017 PMC7197389

[54] B. Qin , J. Yu , S. Nowsheen , M. Wang , X. Tu , T. Liu , H. Li , L. Wang , Z. Lou , Nat. Commun. 2019, 10, 1242. 30886146 10.1038/s41467-019-09175-0 PMC6423285

[55] D. Simsek , G. C. Tiu , R. A. Flynn , G. W. Byeon , K. Leppek , A. F. Xu , H. Y. Chang , M. Barna , Cell 2017, 169, 1051. 28575669 10.1016/j.cell.2017.05.022 PMC5548193

[56] M. S. Nahorski , S. Maddirevula , R. Ishimura , S. Alsahli , A. F. Brady , A. Begemann , T. Mizushima , F. J. Guzman‐Vega , M. Obata , Y. Ichimura , H. S. Alsaif , S. Anazi , N. Ibrahim , F. Abdulwahab , M. Hashem , D. Monies , M. Abouelhoda , B. F. Meyer , M. Alfadhel , W. Eyaid , M. Zweier , K. Steindl , A. Rauch , S. T. Arold , C. G. Woods , M. Komatsu , F. S. Alkuraya , Brain 2018, 141, 1934. 29868776 10.1093/brain/awy135 PMC6022668

[57] C. Mussolino , M. della Corte , S. Rossi , F. Viola , U. Di Vicino , E. Marrocco , S. Neglia , M. Doria , F. Testa , R. Giovannoni , M. Crasta , M. Giunti , E. Villani , M. Lavitrano , M. L. Bacci , R. Ratiglia , F. Simonelli , A. Auricchio , E. M. Surace , Gene Ther. 2011, 18, 637. 21412286 10.1038/gt.2011.3 PMC3131697

[58] J. W. Bainbridge , A. J. Smith , S. S. Barker , S. Robbie , R. Henderson , K. Balaggan , A. Viswanathan , G. E. Holder , A. Stockman , N. Tyler , S. Petersen‐Jones , S. S. Bhattacharya , A. J. Thrasher , F. W. Fitzke , B. J. Carter , G. S. Rubin , A. T. Moore , R. R. Ali , N. Engl. J. Med. 2008, 358, 2231. 18441371 10.1056/NEJMoa0802268

[59] S. Russell , J. Bennett , J. A. Wellman , D. C. Chung , Z. F. Yu , A. Tillman , J. Wittes , J. Pappas , O. Elci , S. McCague , D. Cross , K. A. Marshall , J. Walshire , T. L. Kehoe , H. Reichert , M. Davis , L. Raffini , L. A. George , F. P. Hudson , L. Dingfield , X. Zhu , J. A. Haller , E. H. Sohn , V. B. Mahajan , W. Pfeifer , M. Weckmann , C. Johnson , D. Gewaily , A. Drack , E. Stone , et al., Lancet 2017, 390, 849. 28712537 10.1016/S0140-6736(17)31868-8 PMC5726391

[60] E. R. Burnight , J. C. Giacalone , J. A. Cooke , J. R. Thompson , L. R. Bohrer , K. R. Chirco , A. V. Drack , J. H. Fingert , K. S. Worthington , L. A. Wiley , R. F. Mullins , E. M. Stone , B. A. Tucker , Prog. Retinal Eye Res. 2018, 65, 28. 10.1016/j.preteyeres.2018.03.003 PMC8210531 29578069

[61] W. H. Wu , Y. T. Tsai , S. Justus , T. T. Lee , L. Zhang , C. S. Lin , A. G. Bassuk , V. B. Mahajan , S. H. Tsang , Mol. Ther. 2016, 24, 1388. 27203441 10.1038/mt.2016.107 PMC5023380

[62] J. Ran , M. Liu , J. Feng , H. Li , H. Ma , T. Song , Y. Cao , P. Zhou , Y. Wu , Y. Yang , Y. Yang , F. Yu , H. Guo , L. Zhang , S. Xie , D. Li , J. Gao , X. Zhang , X. Zhu , J. Zhou , Dev. Cell 2020, 53, 287. 32275885 10.1016/j.devcel.2020.03.010

