# Supplementary material for: KIF11 UFMylation Maintains Photoreceptor Cilium Integrity and Retinal Homeostasis
Source: Adv Sci (Weinh). 2024 Apr 26;11(25):2400569. doi: 10.1002/advs.202400569 (PMC11220646; doi:10.1002/advs.202400569)
Supplement: Supplementary file 1 — Supporting Information [file ADVS-11-2400569-s001.pdf]

ADVANCED SCIENCE Open Access

Supporting Information

for *Adv. Sci.*, DOI 10.1002/advs.202400569

KIF11 UFMylation Maintains Photoreceptor Cilium Integrity and Retinal Homeostasis

Jie Ran, Guizhi Guo, Sai Zhang, Yufei Zhang, Liang Zhang, Dengwen Li, Shian Wu, Yusheng Cong, Xiaohong Wang, Songbo Xie, Huijie Zhao, Hongbin Liu, Guangshuo Ou, Xueliang Zhu, Jun Zhou* and Min Liu**

Supporting Information

KIF11 UFMylation maintains photoreceptor cilium integrity and retinal homeostasis

Jie Ran[1]*, Guizhi Guo[1], Sai Zhang[1], Yufei Zhang[1], Liang Zhang[1], Dengwen Li[2], Shian Wu[2], Yusheng Cong[3], Xiaohong Wang[4], Songbo Xie[1], Huijie Zhao[1], Hongbin Liu[5], Guangshuo Ou[6], Xueliang Zhu[7], Jun Zhou[1,2]*, Min Liu[8]*

[1]Center for Cell Structure and Function, Shandong Provincial Key Laboratory of Animal Resistance Biology, Haihe Laboratory of Cell Ecosystem, College of Life Sciences, Shandong Normal University, Jinan 250014, China.
[2]Department of Genetics and Cell Biology, State Key Laboratory of Medicinal Chemical Biology, College of Life Sciences, Nankai University, Tianjin 300071, China.
[3]Key Laboratory of Aging and Cancer Biology of Zhejiang Province, Institute of Aging Research, School of Medicine, Hangzhou Normal University, Hangzhou 310036, China.
[4]Department of Pharmacology, Tianjin Key Laboratory of Inflammation Biology, School of Basic Medical Sciences, Tianjin Medical University, Tianjin 300070, China.
[5]Center for Reproductive Medicine, Cheeloo College of Medicine, Key Laboratory of Reproductive Endocrinology of Ministry of Education, Shandong University, Jinan, 250014, China
[6]Tsinghua-Peking Center for Life Sciences, Ministry of Education Key Laboratory for Protein Science, School of Life Sciences, Tsinghua University, Beijing 100084, China.
[7]State Key Laboratory of Cell Biology, CAS Center for Excellence in Molecular Cell Science, Shanghai Institute of Biochemistry and Cell Biology, Chinese Academy of Sciences, Shanghai 200031, China.
[8]Laboratory of Tissue Homeostasis, Haihe Laboratory of Cell Ecosystem, Tianjin 300462, China.

*Correspondence: jran@sdnu.edu.cn (J.R.), junzhou@sdnu.edu.cn (J.Z.), minliu@nankai.edu.cn (M.L.)

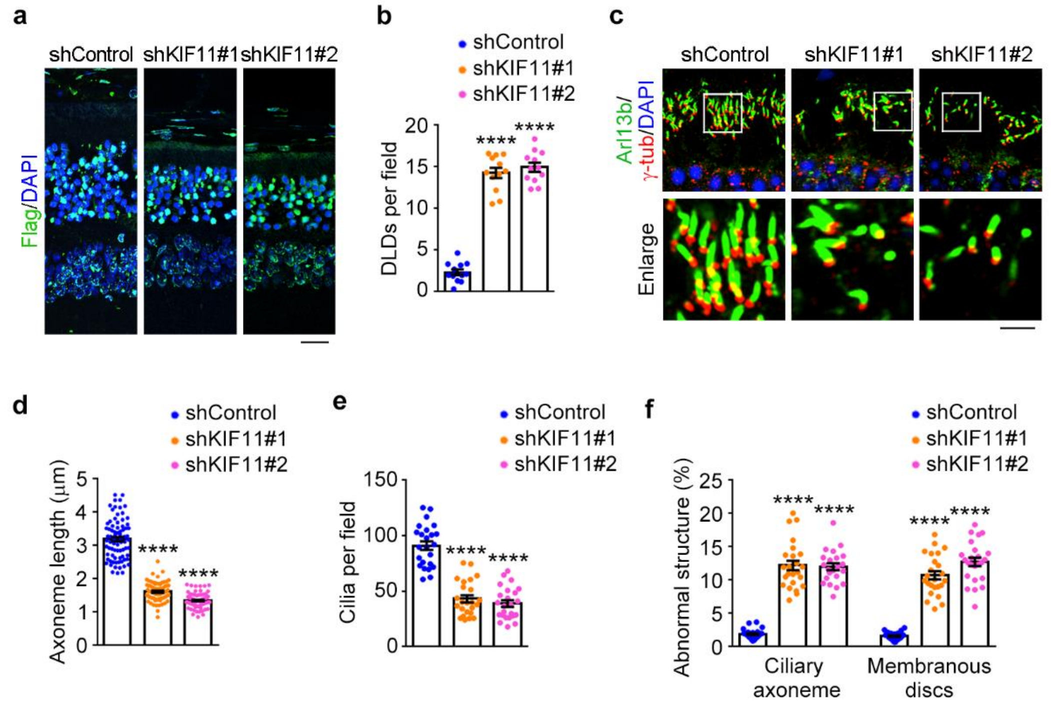

Figure S1. KIF11 knockdown induces defects in the photoreceptor cilium.

a) Immunofluorescence images of the retinas of control or KIF11-shRNA adenovirus-injected mice. Scale bar, 10 μm. b) Quantification of the number of DLDs per field in the retinas of control or KIF11-shRNA adenovirus-injected mice (n = 12 mice of three independent experiments). c-e) Immunofluorescence images (c) and quantification of ciliary axoneme length (d, n = 80 fields from 12 mice of three independent experiments) and ciliary density (e, n = 24 eyes from 12 mice of three independent experiments) in the retinas of control or KIF11-shRNA adenovirus-injected mice. Scale bar, 3μm. f) Quantification of abnormal structures of the ciliary axoneme and membranous discs in the retinas of control or KIF11-shRNA adenovirus-injected mice (n = 24 eyes from 12 mice of three independent experiments).

Data are presented as mean ± SEM. ****$p < 0.0001$.

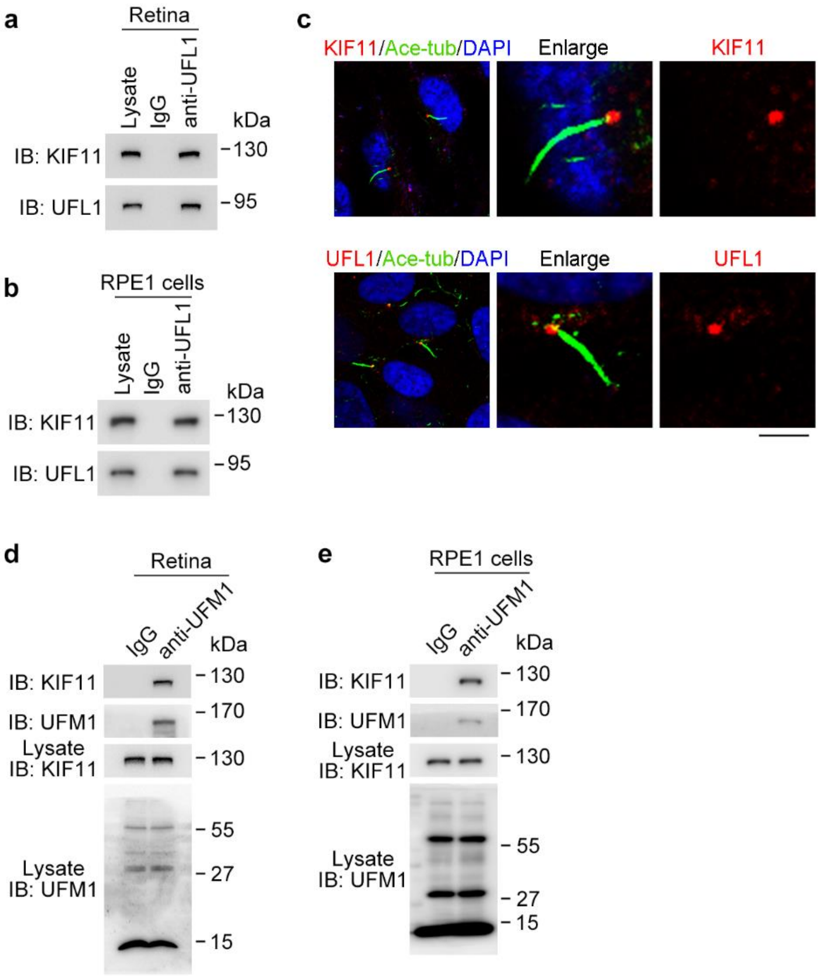

Figure S2. Analysis of KIF11 UFMylation.
a, b) Immunoprecipitation and immunoblotting showing the interaction between endogenous KIF11 and UFL1 in mouse retinas and RPE1 cells. c) Immunofluorescence staining of KIF11, UFL1, and acetylated tubulin (Ace-tub) in RPE1 cells. Scale bar, 2 μm. d, e) Endogenous KIF11 UFMylation was analyzed by immunoprecipitation with the anti-UFM1 antibody followed by immunoblotting with the anti-KIF11 antibody in mouse retinas (d) and RPE1 cells (e).

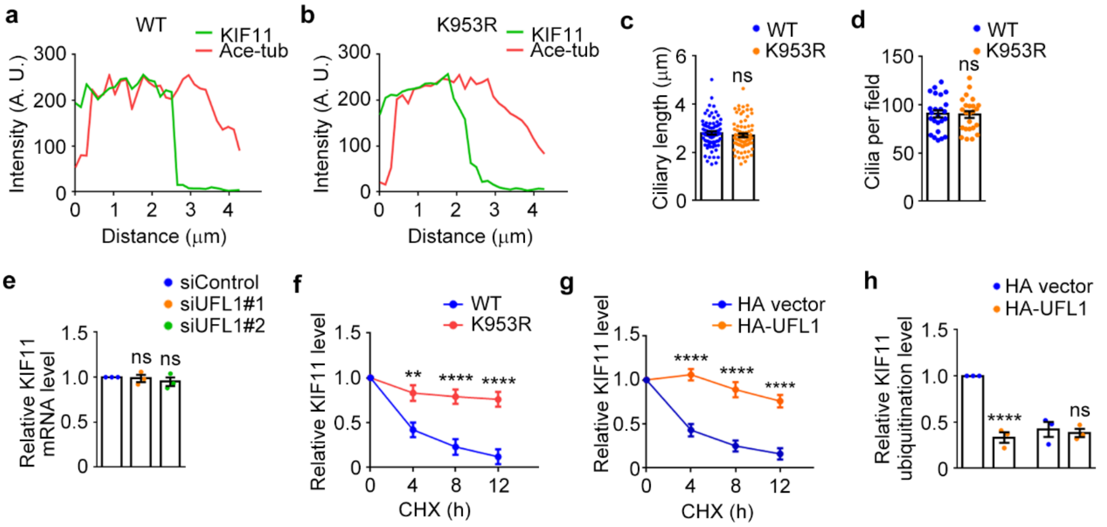

Figure S3. UFL1-mediated KIF11 UFMylation enhances KIF11 protein level.
a, b) Fluorescence intensity of KIF11 and acetylated tubulin along the photoreceptor cilium (from the basal body to the tip of the cilium) in retinas from wild-type and K953R knock-in mice (Related to Figure 4b). c, d) Quantification of the length (c, n = 80 fields from 12 mice of three independent experiments) and intensity (d, n = 24 eyes from 12 mice of three independent experiments) of ciliary axonemes in retinas from wild-type and K953R knock-in mice (Related to Figure 4b). e) Quantitative RT-PCR analysis of relative KIF11 mRNA levels in cells transfected with control or UFL1 siRNAs (n = 3 independent experiments). f) Quantification of relative GFP-KIF11 protein levels in HEK293T cells transfected with GFP-KIF11 wild-type or the K953R mutant (Related to Figure 4e, n = 3 independent experiments). g) Quantification of relative KIF11 protein levels in HEK293T cells transfected with HA vector or HA-UFL1 plasmid (Related to Figure 4h, n = 3 independent experiments). h) Quantitation of relative KIF11 ubiquitination level in cells transfected with HA-UFM1, His-Myc-ubiquitin, GFP-KIF11, or GFP-KIF11-K953R together with HA vector or HA-UFL1 (Related to Figure 4o, n = 3 independent experiments).
Data are presented as mean ± SEM. $^{**}p < 0.01$, $^{****}p < 0.0001$; ns, not significant.

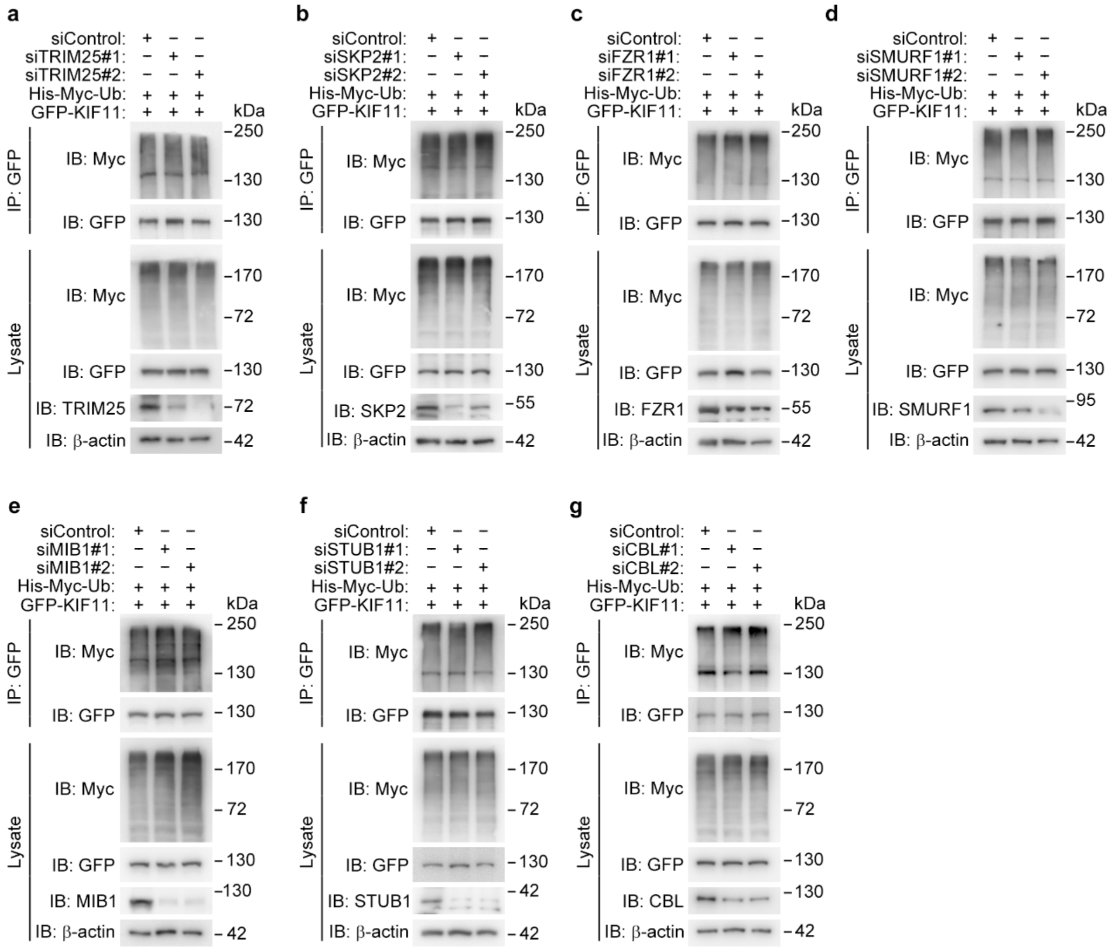

Figure S4. Examination of KIF11 ubiquitination.

a-g) Analysis of KIF11 ubiquitination by immunoprecipitation with the anti-GFP antibody followed by immunoblotting with the anti-Myc antibody in HEK293T cells transfected with GFP-KIF11 and His-Myc-ubiquitin, together with control, TRIM25 (a), SKP2 (b), FZR1 (c), SMURF1 (d), MIB1 (e), STUB1 (f), or CBL (g) siRNAs.

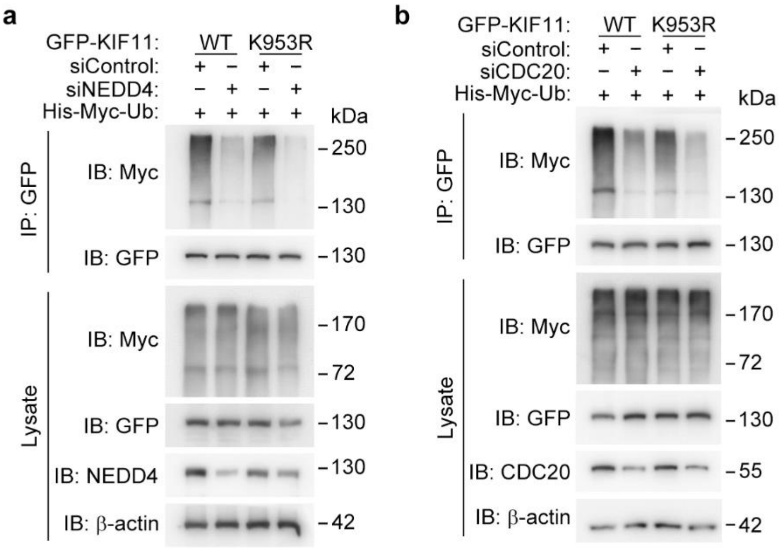

Figure S5. KIF11 UFMylation at K953 does not affect its ubiquitination by NEDD4 or CDC20.

a, b) Analysis of KIF11 and K953R ubiquitination by immunoprecipitation with the anti-GFP antibody followed by immunoblotting with the anti-Myc antibody in HEK293T cells transfected with GFP-KIF11 and His-Myc-ubiquitin, together with control, NEDD4 (a), or CDC20 (b) siRNAs.

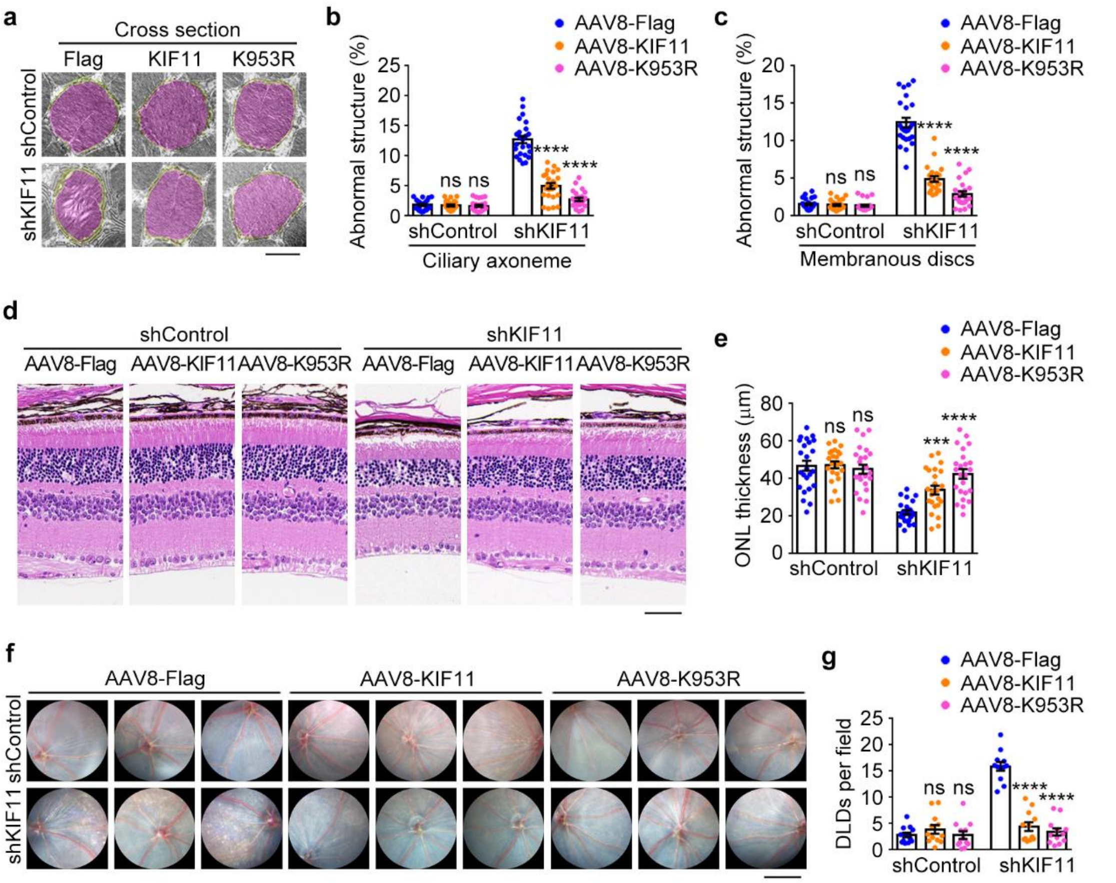

Figure S6. UFMylation plays a crucial role in the maintenance of retinal homeostasis by KIF11.

a) Transmission electron microscopy images of the cross sections of membranous discs in retinas injected with control or KIF11-shRNA adenoviruses, together with AAV-Flag, AAV-KIF11, or AAV-KIF11-K953R. Scale bar, 0.5 μm. b, c) Quantification of abnormal structures of ciliary axonemes (b, n = 24 eyes from 12 mice of three independent experiments) and membranous discs (c, n = 24 eyes from 12 mice of three independent experiments) in retinas injected with control or KIF11-shRNA adenoviruses, together with AAV-Flag, AAV-KIF11, or AAV-KIF11-K953R. d, e) Photomicrographs (d) and quantification (e) of the retinal histology assessed by H&E staining in retinas injected with control or KIF11-shRNA adenoviruses, together with AAV-Flag, AAV-KIF11, or AAV-KIF11-K953R (n = 24 eyes from 12 mice of three independent experiments). Scale bar, 30 μm. f, g) Images of fundus photography (f) and quantification of DLDs per field (g) in retinas injected with control or KIF11-shRNA adenoviruses, together with AAV-Flag, AAV-KIF11, or AAV-KIF11-K953R (n = 12 mice from three independent experiments).

Data are presented as mean ± SEM. ***$p < 0.001$, ****$p < 0.0001$; ns, not significant.
